# Exacerbation of Cisplatin Cellular Toxicity by Regulation of the Human Organic Cation Transporter 2 through Angiotensin II

**DOI:** 10.3390/ijms232415866

**Published:** 2022-12-14

**Authors:** Marta Kantauskaite, Anna Hucke, Beatrice Snieder, Giuliano Ciarimboli

**Affiliations:** 1 Experimental Nephrology, Department of Internal Medicine D, University Hospital Münster, 48149 Münster, Germany; 2 Department of Nephrology, Medical Faculty, University Hospital Düsseldorf, Heinrich-Heine-University Düsseldorf, 40225 Düsseldorf, Germany; 3 Institute of Physiology II, University of Münster, 48149 Münster, Germany

**Keywords:** cisplatin, renin-angiotensin-aldosterone-system, transport, organic cation transporter, regulation, toxicity

## Abstract

Cisplatin (CDDP) is an efficient chemotherapeutic drug, whose use is associated with the development of serious undesired toxicities, such as nephrotoxicity. The human organic cation transporter 2 (hOCT2), which is highly expressed in the basolateral membrane domain of renal proximal tubules seems to play an important role in the development of CDDP nephrotoxicity. The role of angiotensin II (AII) signaling by binding to the AII receptor type 1 (AT1R) in the development and/or progression of CDDP nephrotoxicity is debated. Therefore, in this work, the regulation of hOCT2 activity by AII and its role in the development of CDDP cellular toxicity was investigated. To do this, hOCT2 was overexpressed by viral transduction in Madin–Darby Canine Kidney (MDCK) cells which were cultivated on a filter. This approach allows the separation of an apical and a basolateral membrane domain, which are easily accessible for experimentation. In this system, hOCT2 was mainly localized on the basolateral plasma membrane domain of the cells. The transporter was functional since a specific uptake of the fluorescent organic cation 4-(4-(dimethylamino)styryl)-N-methylpyridinium (ASP^+^) with an affinity (K_m_) of 35 µM was only detectable by the addition of ASP^+^ to the basolateral compartment of hOCT2 expressing MDCK (hOCT2-MDCK) cells. Similarly, CDDP toxicity was evident mainly by CDDP addition to the basolateral compartment of hOCT2-MDCK cells cultivated on a filter. The addition of 1 nM AII stimulated hOCT2 function via PKC activation and worsened CDDP cytotoxicity via binding to AT1R. Therefore, the AII signaling pathway may be implicated in the development and/or progression of CDDP nephrotoxicity. This signaling pathway may be a target for protective interventions for example by blocking AT1R in the kidneys. However, it should be further investigated whether these findings obtained in a cell culture system may have translational relevance for the clinical situation. For toxicity experiments, a 100 µM CDDP concentration was used, which is high but allows us to identify clearly toxic effects due to hOCT2. In summary, down-regulation of hOCT2 activity by the inhibition of the AII signaling pathway may protect against CDDP nephrotoxicity.

## 1. Introduction

Platinum derivatives such as cisplatin and oxaliplatin are important and efficient chemotherapeutic drugs used in the treatment of solid cancers [1]. A major problem of cancer chemotherapy with Platinum derivatives is the development of severe side effects, which can compromise the efficacy of tumor treatment and life quality after cancer. For example, chemotherapy with cisplatin (CDDP), which is considered to be curative against testicular cancer [2], can induce nephrotoxicity [3] and ototoxicity [4], while oxaliplatin treatment causes peripheral neurotoxicity in most patients [5]. Therefore, it seems that platinum derivatives have specific toxicity against selected organs. A possible explanation for these specific toxicities is that these Platinum derivatives interact with transporters for organic cations (OCT), a family of transport proteins that are highly expressed in organs and structures such as the kidneys, the inner ear, and the peripheral root ganglia, which are a target of CDDP and oxaliplatin undesired toxicities. Indeed, it has been demonstrated that CDDP [6,7,8], oxaliplatin [9], the third-generation Platinum derivative picoplatin [10] and the recently synthesized Platinum agent phenanthriplatin [11] are substrates for OCT2. The OCT2 is an OCT subtype, which is highly expressed on the basolateral membrane domain of renal proximal tubules cells [12,13], on the plasma membrane of Corti organ outer hair cells and stria vascularis cells [14], and in peripheral root ganglia [9]. By interacting with OCT2, these Platinum agents may enter these cells and cause specific toxicity. Mutations of hOCT2 may modulate the sensitivity against CDDP toxic effects [15].

Focusing on the kidneys, CDDP is excreted not only by glomerular filtration but also via secretion by proximal tubules [16]. The OCT2 expressed in the basolateral plasma membrane domain of renal proximal tubules’ cells mediates the first step of CDDP renal secretion, the CDDP uptake into the proximal tubules’ cells [17]. In this way, OCT2 plays a prominent role in the development of CDDP nephrotoxicity. The inhibition of OCT2 by substances such as cimetidine and pantoprazole may represent a useful approach to protect cells against undesired toxicity caused by Platinum agents [14,18,19,20,21,22,23] since cancer cells seem to express no or only low levels of this transporter [14]. Interestingly, the activity of OCT2 can be rapidly regulated by several signaling pathways [24,25,26] and by pathological states [27,28,29], suggesting a possible role of transporter regulation for modulating side effects of chemotherapy with Platinum agents. The hOCT2 regulation can be dependent on cell polarization [26], that is the formation of distinct apical and basolateral membrane domains, which is typical for renal epithelial cells.

The signaling pathway associated with angiotensin II (AII), a key member of the renin-angiotensin-aldosterone-system (RAAS), is a potent regulator of renal hemodynamic and tubular function to conserve salt and water, in this way influencing arterial pressure. These AII effects are mediated by its binding to the angiotensin II receptor type 1 (AT1R) [30]. Dysregulation of RAAS can cause hypertension and its pharmacological inhibition, e.g., blocking the AT1R with specific inhibitors such as Losartan is an option to treat hypertension [30]. Among other actions, in the kidneys, AII regulates tubular epithelial cell water and NaCl transport [30]. Interestingly, the role of AII and of AT1R in CDDP-induced renal toxicity is highly debated. Some studies found protection of renal function against CDDP nephrotoxicity using inhibition of AII synthesis or blocking the AT1R with Losartan [31,32,33]. Of note, in rodent models of CDDP nephrotoxicity, the protective effect of AT1R inhibition seems to be evident only in male animals, suggesting a sex-related difference [34]. Conversely, other studies found an exacerbation of CDDP nephrotoxicity by RAAS inhibition [35]. To summarize, hOCT2 accepts CDDP as a substrate and mediates CDDP-induced nephrotoxicity. The activity of hOCT2 can be regulated by many signaling pathways. The extent of CDDP nephrotoxicity may be changed by activation of the AII signaling pathway, which is an important regulator of kidney function.

Therefore, in this work, it was investigated whether OCT2 activity can be regulated by AII and whether such regulation can modulate CDDP cellular toxicity. These studies were performed using Madin–Darby–Canine–Kidney (MDCK) cells, a cellular model, where the cells polarize and form distinct apical and basolateral membrane domains, a feature typical of renal epithelial cells.

## 2. Results

### 2.1. Characterization of MDCK Cells Used in This Study

**Figure 1:**
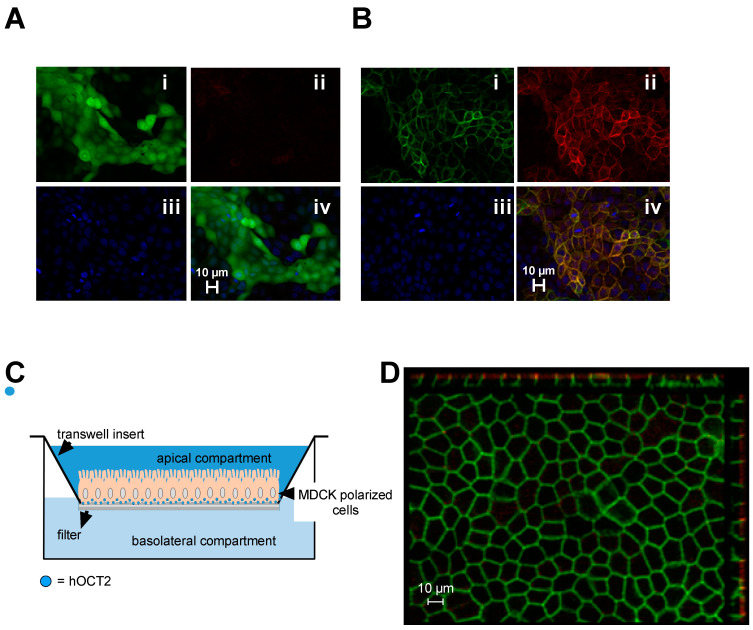
This figure summarizes the cells and experimental setups used in this work. Panel (**A**) shows an immunofluorescence analysis of MDCK cells stably expressing the GFP vector (EV-MDCK cells). Panel (**Ai**) shows the GFP green fluorescence, which is distributed across the entire cell. In panel (**Aii**) no signal is visible when the cells were stained with an antibody against hOCT2. Panel (**Aiii**) shows the nuclei labeling with 4′,6-diamidino-2-phenylindole (DAPI) and panel Aiv is an overlay picture of the three labelings. Panel (**B**) shows an immunofluorescence analysis of MDCK cells stably expressing the hOCT2-GFP (hOCT2-MDCK cells). Panel (**Bi**) shows the GFP green fluorescence, which is mainly associated with the plasma membrane. Panel (**Bii**) shows the staining of the cells with an antibody against hOCT2 (red). Panel (**Biii**) shows the nuclei labeling with DAPI and panel (**Biv**) is an overlay picture of the three labelings, where co-localization of GFP (green) and hOCT2 (red) staining is evident (yellow color). In both panels (**Aiv**,**Biv**) a 10 µm scale bar is shown. Panel (**C**) shows a schematic representation of the MDCK cells growing on filters. Growing on a filter, MDCK cells form a monolayer, which separates an apical and a basolateral compartment. Both compartments are accessible to experimental maneuvers. In panel (**C**) the small blue dots in the MDCK cells represent the hOCT2 cellular distribution. Panel (**D**) shows a representative immunofluorescence analysis of MDCK-hOCT2 cells growing on filter. The green labeling represents the GFP signal of the hOCT2-GFP construct and the red color labels the zona occludens 1 protein (ZO1), which is an important component of the tight junctions. On the projections, orthogonal views of z-stack images are shown. A 10 µm scale bar is also shown. The image shows a clear basolateral localization of hOCT2-GFP.

An empty vector containing a sequence coding for green fluorescent protein (GFP) (EV) or a hOCT2-GFP construct was inserted in MDCK cells by viral transduction to generate EV- and hOCT2-MDCK cells. While in EV-MDCK cells the GFP fluorescence was distributed across the cells (Figure 1, panel Ai), in hOCT2-MDCK cells it was mainly localized in the plasma membrane (Figure 1, panel Bi). Labeling with an antibody against hOCT2 did not show any signal in EV-MDCK cells (Figure 1, panel Aii). In hOCT2-MDCK cells, the GFP fluorescence colocalized with the antibody labeling of hOCT2, confirming the correspondence of the GFP fluorescence with hOCT2 (Figure 1, panel Biv). In Figure 1, the experimental setup used in this study, where MDCK are cultivated on a transwell permeable support, which provides independent access to both the apical and basolateral side of the monolayer, is illustrated in panel C. Both the apical and basolateral compartments are accessible for experimentation. The immunofluorescence analysis of hOCT2-MDCK cells growing on filters is shown in panel D of Figure 1, where the basolateral hOCT2 localization is evidenced by the hOCT2-associated GFP fluorescence.

**Figure 2:**
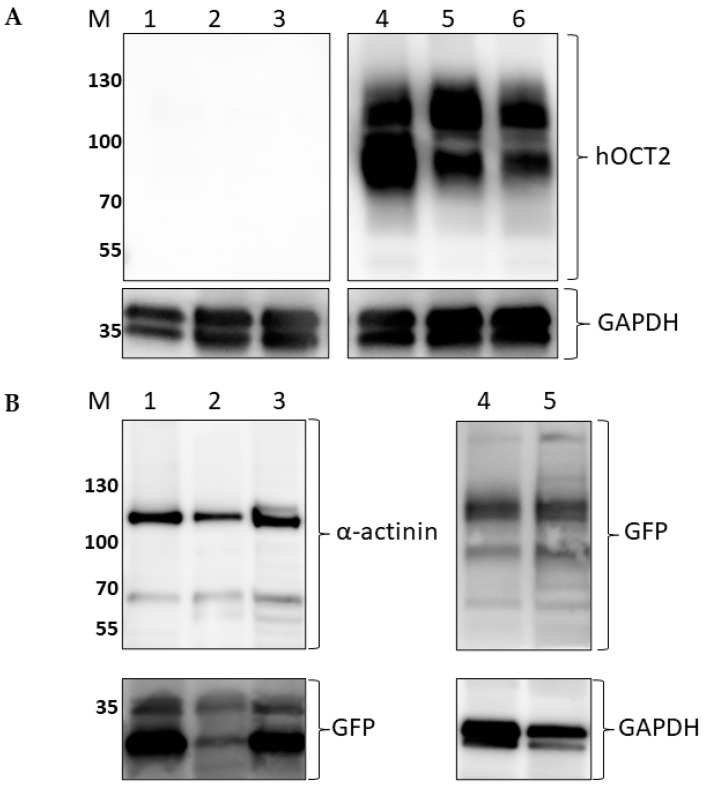
This figure shows a Western blot analysis of hOCT2 expression in the MDCK cells stably expressing hOCT2-GFP (hOCT2-MDCK) or the GFP empty vector (EV-MDCK). Panel (**A**) shows the hOCT2 labeling in lysates from EV (lanes 1–3)- and hOCT2 (lanes 4–6)-MDCK cells. Only lysates from hOCT2-MDCK cells showed signals corresponding to hOCT2 at a molecular weight of around 90 and 120 kDa, while no band was visible in lysates from EV-MDCK cells. The labeling of hOCT2 in hOCT2-MDCK cells resulted in two bands, which were already characterized as a glycosylated and a non-glycosylated form of hOCT2 [36]. The lower part of panel (**A**) shows the signal deriving from labeling with an antibody against GAPDH as a loading control. Panel (**B**) shows the GFP labeling in lysates from EV (lanes 1–3)- and hOCT2 (lanes 4–5)-MDCK cells. Lysates from EV-MDCK cells (lanes 1–3) show GFP signals below the 35 kDa molecular weight, probably corresponding to cytosolic GFP and its degradation products. Lysates from hOCT2-MDCK cells (lanes 4–5) show GFP signals at around 90 and 120 kDa molecular weight, confirming the presence of hOCT2-GFP in this preparation. α-actinin and GAPDH were used as a loading control for EV-MDCK (lanes 1–3) and hOCT2-MDCK (lanes 4–5) cells, respectively. Antibodies against GAPDH are well known to be able to detect also a band below 36 kDa, which probably corresponds to an isoform or a spliced product of GAPDH [37,38]. M indicates the lane containing the molecular weight markers, which are given as kDa.

The hOCT2 expression in EV- and hOCT2-MDCK cells was further investigated by Western blot analysis. As evident from the inspection of Figure 2, only the hOCT2-MDCK cells express hOCT2. Both detections with antibodies against hOCT2 and with antibodies against GFP revealed the transporter expression (Figure 2, lanes 4–6 in panel A and 4–5 in panel B).

### 2.2. Characterization of Organic Cation Transport in MDCK Cells Stably Expressing GFP or hOCT2-GFP

**Figure 3:**
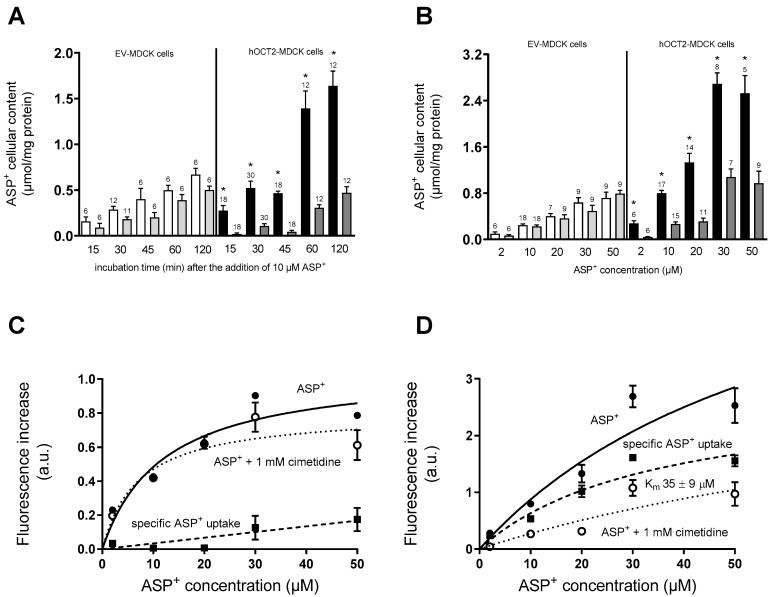
The graph shows the characteristics of ASP^+^ transport after addition of this fluorescent hOCT2 substrate to the basolateral compartment of EV- and hOCT2-MDCK cells growing on a filter. Panel (**A**) shows the time-dependent ASP^+^ cellular content after incubation with 10 µM ASP^+^ for 15–120 min. In EV-MDCK cells, a small increase in cellular ASP^+^ content over time was measured (white columns). However, this uptake was not significantly inhibited by co-incubation with 1 mM cimetidine (grey columns). Only hOCT2-MDCK cells showed an ASP^+^ accumulation (closed columns), which was significantly inhibited in the presence of 1 mM cimetidine (grey columns). The first incubation time, where ASP^+^ cellular content of hOCT2-MDCK cells was clearly higher than in EV-MDCK cells was 60 min. Therefore, in further experiments, this incubation time was used. Panel (**B**) shows the ASP^+^ cellular content after incubation of EV- and hOCT2-MDCK cells growing on a filter with 2–50 µM ASP^+^ for 60 min from the basolateral compartment. Again, only hOCT2-MDCK cells showed an ASP^+^ accumulation (closed columns), which was significantly inhibited in the presence of 1 mM cimetidine (grey columns). The numbers on the columns show the replicates measured in at least three independent experiments. The star (*) shows a statistically significant difference (*p* < 0.05, unpaired *t*-test) compared to correspondent experiments performed using a large excess of cimetidine (1 mM). Panels (**C**,**D**) show the ASP^+^ cellular content after addition to the basolateral compartment of EV- and hOCT2-MDCK cells, respectively, of 2–50 µM ASP^+^ for 60 min. Measurements (4–17 replicates for every concentration, measured at least in three independent experiments) were performed in the presence (open circles, unspecific ASP^+^ accumulation, dotted lines) or not (closed circles, total ASP^+^ accumulation, solid lines) of 1 mM cimetidine to determine the part of ASP^+^ cellular accumulation, which is probably not mediated by OCT2. By subtracting the unspecific from the total ASP^+^ accumulation, the “specific” (hOCT2-mediated, dashed lines) transport was determined. Only for hOCT2-MDCK cells (panel (**D**)) this transport reached saturation, allowing us to calculate its K_m_ (35 ± 9 µM).

The transport characteristics of the fluorescent organic cation 4-(4-dimethylaminostyryl)-N-methylpyridinium (ASP^+^) in EV- and hOCT2-MDCK cells cultured on a filter were determined at different times after the addition of 10 µM ASP^+^ to the basolateral compartment of the transwell in the presence or not of a large excess of cimetidine (1 mM). The large excess of cimetidine is used to completely block the ASP^+^ transport by hOCT2 in this way determining the cellular ASP^+^ accumulation, which is not mediated by hOCT2 (unspecific uptake). ASP^+^ is a well-known fluorescent substrate of transporters for organic cations, which allows for measuring the transporter activity [39,40,41]. As shown in panel A of Figure 3, only hOCT2-MDCK cells showed an inhibitable ASP^+^ uptake at each investigated time point. After 1 h incubation, a striking difference in the accumulation of ASP^+^ was observed between EV- and hOCT2-MDCK cells. Therefore, in further experiments, the cellular ASP^+^ content was measured after 1 h incubation of MDCK cells. As shown in panel B of Figure 3, hOCT2-MDCK cells showed a cimetidine inhibitable ASP^+^ uptake at every tested concentration, in contrast to EV-MDCK cells.

As the next step, the saturation curve of ASP^+^ accumulation in cellular lysates from EV- (panel C of Figure 3) and hOCT2-MDCK cells (panel D of Figure 3) after 1 h incubation with increasing ASP^+^ concentrations (2–50 µM) was determined. EV-MDCK cells showed under these conditions no saturable specific (total minus “unspecific”) ASP^+^ accumulation and, therefore, no kinetic parameter could be determined with precision. Conversely, hOCT2-MDCK cells showed a saturable specific ASP^+^ uptake with a K_m_ value of 35 µM. Therefore, further transport experiments were performed using incubation with 20 µM ASP^+^ for 1 h.

**Figure 4:**
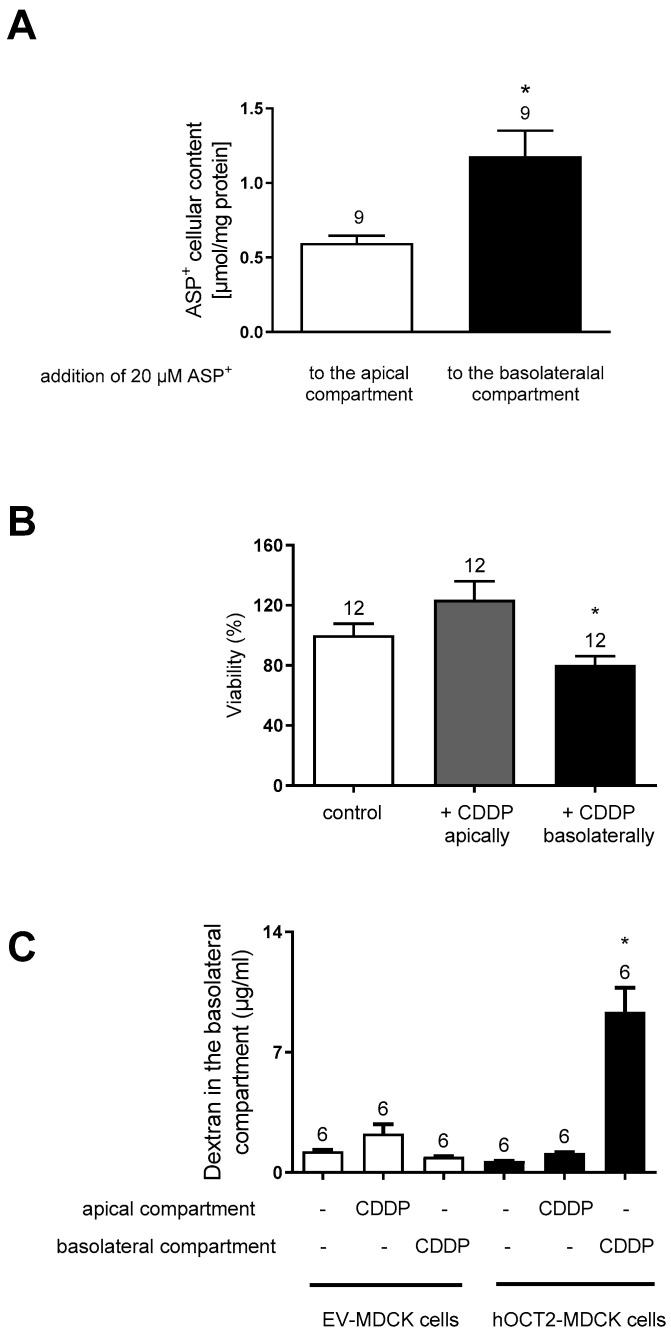
This figure shows the effects of MDCK cells treatment from the apical or basolateral compartment. Panel (**A**) shows the cellular accumulation of ASP^+^ measured after 60 min incubation with 20 µM ASP^+^, which was added to the apical or the basolateral compartment of hOCT2-MDCK cells growing on a filter. Addition to the basolateral compartment resulted in a significantly higher ASP^+^ cellular accumulation than addition to the apical compartment (*, *p* < 0.05, unpaired *t*-test). The numbers on the columns show the replicates measured in at least three independent experiments. Panel (**B**) shows toxicity of 100 µM CDDP addition to the apical or to the basolateral compartment of hOCT2-MDCK cells growing on a filter. After 4 h incubation with 100 µM CDDP from the apical or basolateral compartment, CDDP was replaced by a Ringer-like solution and the cells were further incubated for 48 h before determination of cell viability with a 3-(4,5-dimethylthiazol-2-yl)-2,5-diphenyltetrazolium (MTT) test. A significant decrease in cell viability was observed only after CDDP incubation from the basolateral side. The numbers on the columns show the replicates measured in at least three independent experiments. The star (*) shows a statistically significant difference (*p* < 0.05, ANOVA test with Tukey multiple comparison) compared to control experiments and experiments, where CDDP was added to the apical membrane domain. Panel (**C**) shows the results of permeability measurements performed using FITC-dextran 70 kDa. CDDP (100 µM) was added to the apical or basolateral compartment of EV- or hOCT2-MDCK cells growing on a filter. After 4 h, CDDP was replaced by medium without phenol red and 1 mg/mL dextran was added to the apical compartment. The concentration of dextran in the basolateral compartment was measured after 10, 30, 180, and 240 min and after 72 h (only these results are illustrated in the figure). Only addition of CDDP to the basolateral compartment of hOCT2-MDCK cells caused after 72 h a significant increase in dextran concentration in the basolateral compartment. The numbers on the columns show the replicates measured in at least 3 independent experiments. The star (*) shows a statistically significant difference (*p* < 0.05, ANOVA test with Tukey multiple comparisons) compared to control experiments and experiments, where CDDP was added to the apical compartment of the transwell.

Next, the ASP^+^ transport and CDDP toxicity were investigated in hOCT2-MDCK cells by adding these substances to the apical or basolateral compartment of the transwell. As shown in panel A of Figure 4, the addition of 20 µM ASP^+^ to the basolateral compartment of hOCT2-MDCK cells growing on the filter resulted in a higher accumulation of the fluorescent tracer than in experiments performed starting the 1 h incubation period by adding ASP^+^ to the apical compartment, confirming the functional importance of hOCT2 presence in the basolateral plasma membrane. The hOCT2 localization on the basolateral plasma membrane domain is also important in determining CDDP cellular toxicity, which, as shown in panel B of Figure 4, was evident only upon the addition of CDDP to the basolateral compartment. The importance of hOCT2 expression for CDDP toxic cellular effects is also shown in panel C of Figure 4, where an increase in the permeability of the cell layer for 70 kDa dextran as an index of cellular toxicity was only measurable upon incubation of hOCT2-MDCK cells with CDDP from the basolateral compartment.

**Figure 5:**
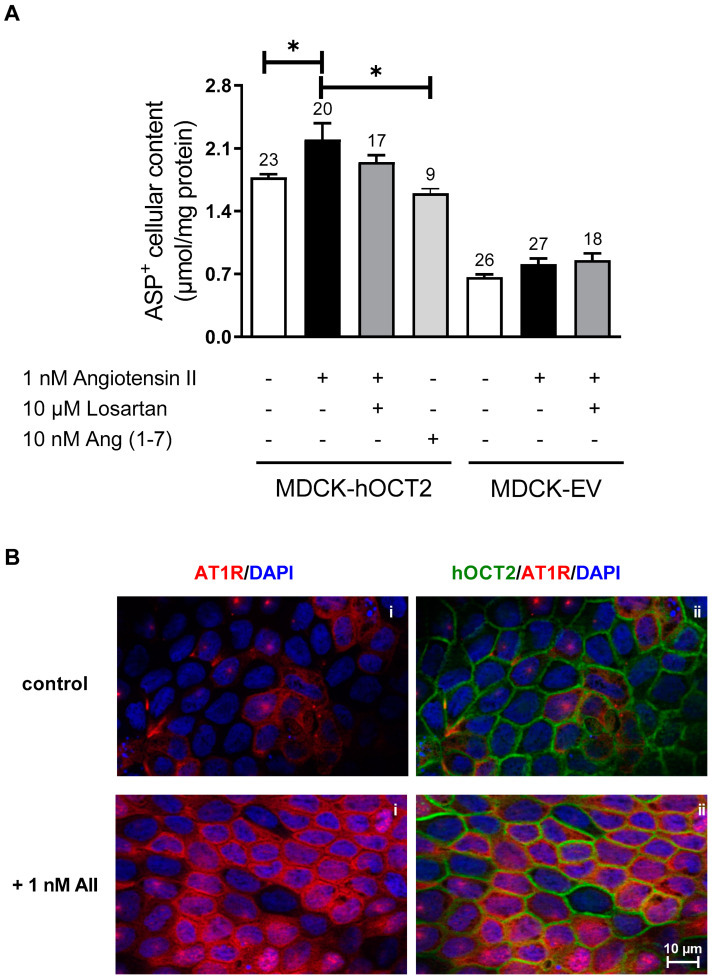
Effects of AII addition to the basolateral compartment of MDCK cells cultivated on filter on basolateral ASP^+^ transport. Panel (**A**) shows the ASP^+^ cellular content after 1 h incubation of EV- or hOCT2-MDCK with 20 µM ASP^+^ in the presence or not of 1 nM AII alone or together with 10 µM Losartan. The effect of 1 h incubation with 10 nM Ang (1−7) on basolateral ASP^+^ transport in hOCT2-MDCK cells is also shown. Incubation with AII significantly increased ASP^+^ cellular content only in experiments with hOCT2-MDCK cells. The numbers on the columns show the replicates measured in at least 3 independent experiments. The star (*) shows a statistically significant difference (*p* < 0.05, ANOVA test with Tukey’s multiple comparisons) compared to control experiments and to experiments with Ang (1−7). Panel (**B**) shows an example of immunofluorescence analysis of AT1R (red) and hOCT2 (green) distribution in dependence from AII incubation in hOCT2-MDCK cells. Nuclei are stained with DAPI in blue. Under control conditions, AT1R seems to localize both in intracellular compartments and partially also in the plasma membrane (control, panel (**i**), red color), while hOCT2 is mainly present in the plasma membrane (control, panel (**ii**), green color). Addition of 1 nM AII for 1 h to the basolateral compartment induces a strong translocation of AT1R to the plasma membrane (+ 1nM AII, panel (**i**), red color), where it partially co-localizes with hOCT2 (+ 1nM AII, panel (**ii**), green color; the yellow color shows an AT1R-hOCT2 co-localization).

Since hOCT2-MDCK cells cultivated on filter seem to be a suitable model to study the properties of hOCT2 in an environment resembling the physiological situation, where hOCT2 is localized in the basolateral plasma membrane domain, the regulation of hOCT2 by AII was investigated in this setting. Incubation of hOCT2-MDCK cells from the basolateral compartment with 1 nM AII, a concentration in the affinity range for AT1R [42], significantly stimulated hOCT2 activity (Figure 5, panel A). This effect seems to be AT1R-specific since it was suppressed by co-incubation with the AT1R inhibitor Losartan at a concentration of 10 µM (according to previous in vitro studies, this Losartan concentration acts specifically on AT1R and the serum Losartan concentration in patients reaches low µM values, depending on the doses [43,44]) and was not present under incubation with Ang (1–7), a heptapeptide formed from angiotensin I and II, which has opposite actions compared to those of AII [45]. Interestingly, MDCK cells show an endogenous expression of AT1R in an intracellular compartment (Figure 5, panel B). AT1R translocates to the plasma membrane under incubation with AII, and partially co-localizes with hOCT2, as shown in Figure 3, panel B. The endogenous AT1R expression in MDCK cells and its translocation to the plasma membrane by incubation with 1 nM AII have already been described in [46].

### 2.3. Pathways Possibly Involved in the Regulation of hOCT2 by AII

**Figure 6:**
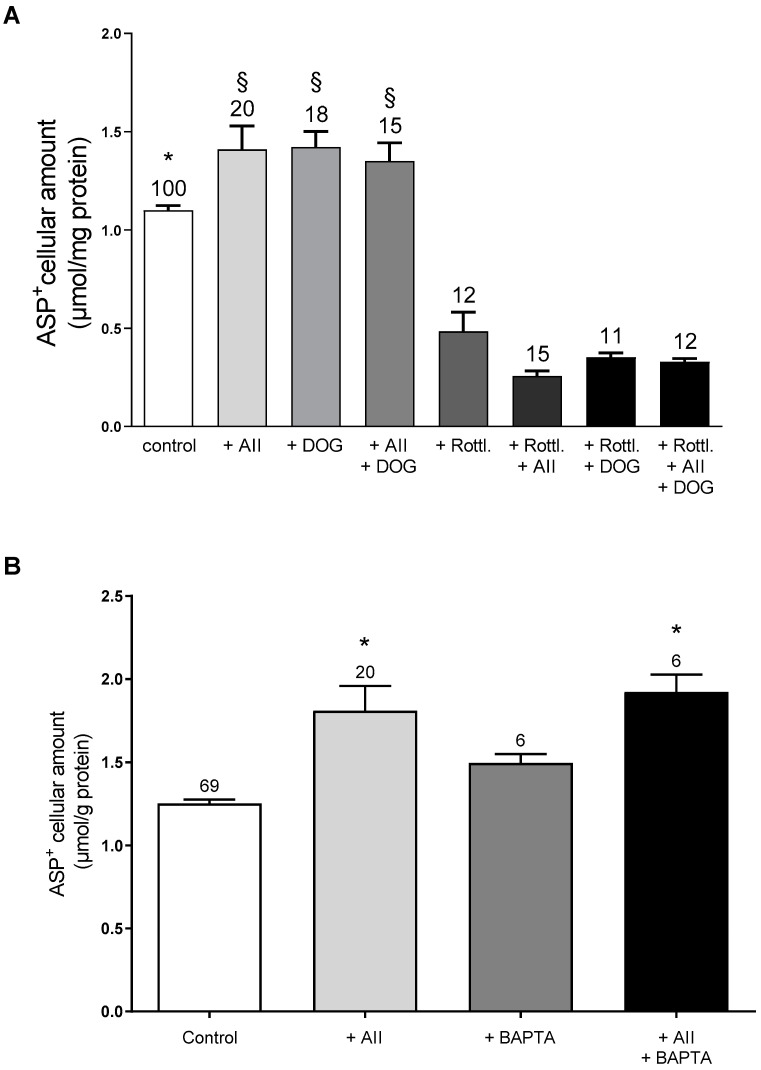
Possible downstream pathways involved in hOCT2 regulation by 1 nM AII in hOCT2-MDCK cells growing on a filter. All substances were added to the basolateral compartment and their influence on cellular ASP^+^ accumulation as a marker of hOCT2 function was evaluated. Panel (**A**) shows the effect of PKC activation or inhibition with 1 µM DOG or 40 µM rottlerin, respectively, on stimulation of hOCT2 by AII. DOG stimulated hOCT2 activity and its addition to AII did not further increased hOCT2 function. Rottlerin alone inhibited hOCT2 activity and completely suppressed the effects of DOG and AII on cellular ASP^+^ accumulation. The numbers on the columns show the replicates measured in at least 3 independent experiments. In panel (**A**) the star (*) shows a statistically significant difference (*p* < 0.05, ANOVA test with Tukey’s multiple comparisons) compared to control experiments, while § shows a statistically significant difference (*p* < 0.05, ANOVA test with Tukey’s multiple comparisons) compared to all other experiments except + AII, + DOG, and +AII + DOG. Panel (**B**) shows the effect of Ca^2+^ chelation with 5 µM BAPTA-AM on stimulation of hOCT2 by AII. Addition of BAPTA-AM did not change the regulation of hOCT2 by AII. The numbers on the columns show the replicates measured in at least three independent experiments. In panel (**B**) the star (*) shows a statistically significant difference (*p* < 0.05, ANOVA test with Tukey’s multiple comparisons) compared to control experiments and to experiments with BAPTA-AM alone.

The binding of AII to AT1R is known to activate a G protein-dependent signaling pathway, which involves heterotrimeric G proteins, including Gq/11, G12/13, and Gi, and a subsequent second messenger including inositol trisphosphate (IP3), diacylglycerol (DAG), phospholipase C (PLC), and PKC. PLC stimulates IP3-mediated Ca^2+^ release and the subsequent activation of other kinases (for a detailed review of the AII signaling pathway please see: [47]). In an attempt to identify the AT1R downstream pathways involved in the AII regulation of hOCT2 activity, the effect of AII on hOCT2 was measured under stimulation/inhibition of PKC using 1,2-dioctanoyl-sn-glycerol (DOG), a cell-permeable activator of PKC, or rottlerin, a cell-permeable specific PKC inhibitor. Moreover, the involvement of Ca^2+^ was investigated using 1,2-bis(2-aminophenoxy)ethane-N,N,N′,N′-tetraacetic acid tetrakis (acetoxymethyl ester) (BAPTA-AM), a cell-permeable selective Ca^2+^ chelator. As shown in panel A of Figure 6, the addition of 1 µM DOG to the ASP^+^ solution in the basolateral compartment significantly increased ASP^+^ cellular accumulation. However, DOG did not further augment the transport of ASP^+^ when administered together with AII, suggesting a maximal PKC activation by these substances. Furthermore, the addition of 40 µM rottlerin, a concentration able to inhibit most PKC subtypes, clearly suppressed both the AII and DOG stimulation of hOCT2-mediated ASP^+^ transport. Interestingly, rottlerin alone strongly decreased ASP^+^ cellular accumulation, suggesting an endogenous activity of PKC in MDCK cells. Chelation of intracellular Ca^2+^ by the addition of 5 µM BAPTA-AM to the incubation solution did not change the stimulatory effect of AII on hOCT2, pointing to a minor role of Ca^2+^ in AII regulation of hOCT2 in this polarized experimental system.

### 2.4. Influence of AII on CDDP Toxicity

**Figure 7:**
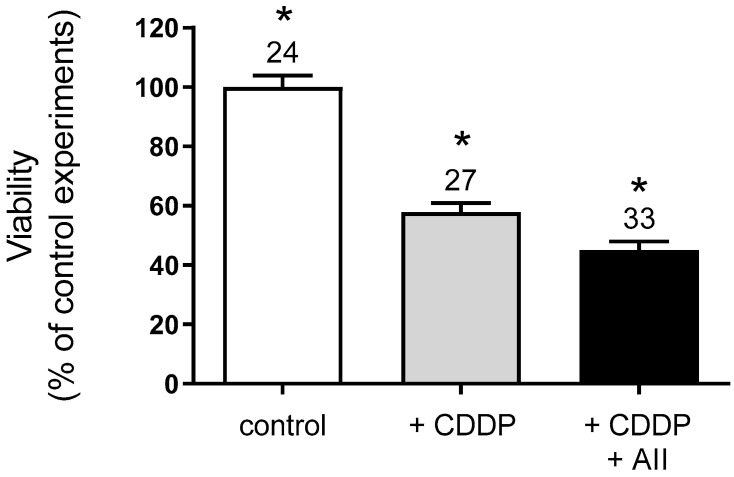
Viability of hOCT2-MDCK cells growing on filter measured by an MTT-test. Cells were incubated for 4 h with medium (white column), or 100 µM CDDP (grey column), or 100 µM CDDP and 1 nM AII (black column) by addition of these substances to the basolateral compartment. Addition of CDDP to the basolateral compartment caused a significant decline in cell viability compared to control experiments (white column). This effect was even worsened by the addition of AII to the incubation solution. The numbers on the columns show the replicates measured in at least three independent experiments. The star (*) shows a statistically significant difference (*p* < 0.05, ANOVA test with Tukey’s multiple comparisons) compared to the other experiments.

Since AII stimulates hOCT2 activity, the effect of this regulation on CDDP cellular toxicity was investigated using an MTT assay of hOCT2-MDCK cells growing on a filter, which were incubated for 4 h with 100 µM CDDP alone or together with 1 nM AII added to the basolateral compartment. Thereafter, the incubation solution was replaced with cell culture medium and the cells were further cultivated for 72 h before the MTT assay. As shown in Figure 7, CDDP caused a significant decline in cell viability, which was further worsened by the addition of AII.

## 3. Discussion

Platinum agents are important chemotherapeutic drugs that are effective against solid tumors. However, their use is often hampered by the insurgence of unwanted toxicities, which compromise the therapeutic effect and/or life quality of patients after cancer treatment. Some of these anticancer drugs interact with organic cation transporters (OCT), which are expressed in tissues, which indeed are especially sensitive to toxicity from Platinum agents such as cisplatin (CDDP) and oxaliplatin. Therefore, it can be supposed that the interaction between OCT and Platinum derivatives may play a role in determining the unwanted toxicities of anticancer treatment. Since most cancer tissues seem not to express OCT, inhibition of transporter function may be an attractive therapeutic option to reduce the side effects of chemotherapeutic treatment with Platinum agents, without changing their antitumor efficacy. In this work, we investigated the effect of regulation of OCT2, a transporter highly expressed in the kidneys and that has been associated with CDDP nephrotoxicity, on cellular CDDP toxicity. Specifically, regulation by the renin-angiotensin-aldosterone-system (RAAS), which is a potent modulator of renal function, and which has been associated with CDDP nephrotoxicity, was investigated. In particular, the influence of angiotensin II (AII), a central mediator of RAAS action, on OCT2 function and CDDP cellular toxicity was studied. Since OCT2 is highly expressed on the basolateral membrane domain of renal proximal tubular cells, its possible regulation by AII was investigated in Madin–Darby Canine Kidney cells, which were genetically modified to express hOCT2 on the basolateral membrane domains, growing on filters. This in vitro system reproduces important physiological characteristics such as the presence of distinct apical and basolateral membrane domains, which are easily accessible for experimentation (see Figure 1). The hOCT2 expressed in these cells by viral transduction showed a clear basolateral cellular distribution; therefore, the experiments were performed, except when otherwise stated, by adding substrate and modulator to the basolateral compartment. In this experimental setting, the affinity of the transporter for ASP^+^ was 35 µM, reflecting the micromolar range of ASP^+^ affinities for hOCT2 found in other studies using different experimental setups [24,48]. In comparison with MDCK cells which expressed the empty vector, hOCT2 overexpression made the cells more sensitive to CDDP toxicity. Under the experimental conditions used in this study, CCDP toxicity was present only upon CDDP addition to the basolateral compartment, probably because of the presence of a high amount of hOCT2 in this plasma membrane domain.

In the toxicity experiments, a high CDDP concentration was used (100 µM) because in the experimental system used, this CDDP concentration induces strong toxicity in hOCT2-MDCK cells but not in EV-MDCK cells (not shown). In patients treated with CDDP, serum CDDP concentrations up to 25–40 µM can be detected [49,50].

The binding of AII to the AT1R stimulated hOCT2 function probably in a PKC-dependent and Ca^2+^-independent manner. A similar activating effect of AII on transport mediated by OCT was observed in freshly isolated renal mouse proximal tubules [51]. Using other experimental setups, for example, human embryonic kidney cells stably overexpressing hOCT2, such regulation by AII was not observed (Supplementary Figure S1), probably due to the fact that these cells do not express AT1R endogenously or because regulation pathways can be different in strongly polarized cells, such as the MDCK cells [26]. In this study, AII stimulation of hOCT2 activity increased CDDP cellular toxicity and, therefore, may be also involved in the development of CDDP-induced nephrotoxicity. In animal studies, it has been demonstrated that treatment of mice with CDDP increased the circulating AII concentration [52]. AII with the renal AT1R are important mediators for the development of hypertension [53]. Interestingly, the presence of hypertension increases the possibility to develop nephrotoxicity in patients treated with CDDP [54]. Therefore, both 1) pre-existing hypertension with higher circulating AII levels and activation of renal AT1R, and 2) AII concentration increase by CDDP treatment may contribute to the worsening of CDDP toxic effects in the kidneys. Inhibition of AT1R may be a strategy to decrease the possibility to develop CDDP-related nephrotoxicity. Against this hypothesis, a retrospective study in cancer patients [55] suggested an association between low blood pressure and CDDP nephrotoxicity, which, however, was observed only in patients fed with non-solid food. Other experimental studies on this topic resulted in controversial results: performing experiments with rats, a clear association between AII-AT1R signaling and CDDP nephrotoxicity was observed only in male but not in female animals [31]. Moreover, in studies with female rats [56], inhibition of AT1R with Losartan even worsened CDDP-induced nephrotoxicity, probably because of a sex-specific increased renal blood flow induced by AT1R blocking. In rodents, the expression of OCT2 in kidneys appears to be sex-dependent, with higher expression in renal tissue from male animals compared with kidneys from female rats [57]. Therefore, specific regulation of OCT2 by AII/AT1R would more strongly impact male than female rats. In humans, such a sex-dependent expression of OCT2 in kidneys could not be detected [58].

In conclusion, in this work, we showed that AII can stimulate the hOCT2 activity when the transporter is expressed in cells, which polarize and form distinguished apical and basolateral membrane domains. This hOCT2 stimulation increases the cellular toxicity of CDDP, suggesting that in cancer patients treated with CDDP an inhibition of AII signaling may decrease CDDP-induced nephrotoxicity. However, the feasibility of this approach should be systematically investigated for successful and responsible therapy employment.

## 4. Materials and Methods

### 4.1. Cloning of hOCT2-GFP into the Viral Transduction Vector

The full-length human organic cation transporter 2 (Solute Carrier (SLC) 22A2, NM_003058) cloned into the expression vector pRc/CMV (hOCT2-pRC/CMV) was a kind gift by Prof. H. Koepsell (University of Würzburg). The hOCT2 from the hOCT2-pRc/CMV was cloned into the pEGFP-N3 vector (Clontech, Saint-Germain-en-Laye, France) via *XhoI* and *BamHI* sites (using the primers listed in Table 1) to obtain a GFP-tagged hOCT2 (hOCT2-GFP, the tag is at the carboxy-terminus) construct. The insertion of the GFP-tag did not change the transport properties of hOCT2 [36,59]. The hOCT2-GFP construct or GFP alone was inserted into the pQCXIH vector (Clontech) via *NotI* and *PacI* using the primers listed in Table 1.

### 4.2. Generation and Culture of MDCK Cell Lines Expressing hOCT2-GFP or GFP Alone

The full-length hOCT2 tagged with GFP (hOCT2-GFP) or the GFP alone (empty vector, EV) cloned in the expression vector pQCXIH were expressed in MDCK II cells (ECACC 00062107) using a retroviral transduction system, as already described in [26]. Using this approach, a high and stable expression of the transferred constructs can be obtained [60,61,62]. Briefly, the packaging cell line GP2-293 (Retro-X, Clontech) was cultivated in standard Dulbecco’s modified Eagle medium (DMEM, Biochrom, Berlin, Germany) supplemented with 10% fetal calf serum (FCS, Biochrom) and 1% antibiotics (penicillin/streptomycin, Biochrom). A recombinant retrovirus was produced by transfection of GP2-293 cells with a plasmid encoding for glycoprotein of vesicular stomatitis virus and the pQCXIH-construct containing hOCT2-GFP or GFP alone. Next, the virus-containing supernatant was filtered through a sterile 0.45 μm filter unit (Millipore, Schwalbach am Taunus, Germany), and the MDCK cells were infected using one volume of fresh DMEM medium and one volume of the virus-containing filtrate supplemented with polybrene (final concentration 1.5 μg/mL) for 24 h. Thereafter, the virus-containing medium was replaced with fresh medium and cells were regenerated for 24 h. Afterward, cells were selected by hygromycin treatment (400 μg/mL). GFP-positive cells were isolated with a cell sorter and further cultured. MDCK cells expressing hOCT2-GFP (hOCT2-MDCK) or GFP alone (EV-MDCK) were cultured in Minimal Essential Medium Eagle (MEM, Sigma/Merck, Darmstadt, Germany) containing 10% FCS, 2 mM L-glutamine and 1% penicillin/streptomycin. For obtaining a system, where both the apical and basolateral plasma membrane domains were accessible to experimentation, cells were seeded on filters (Greiner Bio-one ThinCert # 662641 filters transparent with a pore diameter of 0.4 µm, Kremsmünster, Austria) and cultivated until confluence was reached. Overexpression of ectopic GFP- or GFP-tagged hOCT2 in the stable cell populations was verified by immunofluorescence and Western blot analysis (Figure 1 and Figure 2). These analyses confirmed the expression of the desired proteins and the strong basolateral expression of hOCT2.

Culture and functional analyses of these cells were approved by the state government Landesumweltamt Nordrhein-Westfalen, Essen, Germany (no. 521.-M-1.14/00).

### 4.3. Immunofluorescence Analysis

For immunofluorescence analysis, MDCK cells growing on filter (Greiner, Kremsmünster, Austria) were washed on both apical and basolateral side with Dulbecco’s phosphate buffered saline (PBS, Biochrom) and fixed with 4% paraformaldehyde solution (PFA) for 10 min. After fixation, the cells were washed three times with PBS and incubated with 0.2% Triton X-100 for 5 min. After extensive washing with PBS, unspecific binding sites were blocked by 60 min incubation with 10% bovine serum albumin (BSA, Sigma/Merck, Darmstadt, Germany) in PBS. Filters were cut and then incubated at 4 °C overnight with anti-hOCT2 (HPA008567, Sigma/Merck, Darmstadt, Germany, diluted 1:100 in 1% BSA in PBS) or anti- zona occludens 1 protein (ZO1-1A12, ThermoFischer, Waltham, USA, 1:100 in 1% BSA) or anti-AT1R antibodies (sc-1173, Santa Cruz Biotechnology, Dallas, TX, USA, 1:100 in 1% BSA). After three washing steps in PBS, the secondary antibody (goat-anti-rabbit or goat-anti-mouse Alexa fluor 594, Invitrogen, Karlsruhe, Germany) at a 1:1000 dilution was incubated for 60 min followed by five more washing steps in PBS. The nuclei were labeled with 4′,6-diamidino-2-phenylindole (DAPI, 1 mg/mL,1:1000 in 1% BSA). After 30 min the cells were washed with PBS and covered with Fluoromount (Sigma/Merck, Darmstadt, Germany) and fluorescence photographs were taken with Observer Z1 with Apotome (Carl Zeiss, Göttingen, Germany) using ZEN software.

### 4.4. Western Blot Analysis

For Western blot analysis, MDCK cells grown to confluency were lysed on ice for 20 min with 300 µL lysis buffer (10 mM TRIS-HCl, pH 7.4) containing a protease inhibitors cocktail (c0mplete, Merck, Darmstadt, Germany, 1 tablet/10 mL buffer). Lysates were vortexed, resuspended, and centrifuged at 10,000 g for 5 min at 4 °C. The supernatant was collected and centrifuged again at 20,500 g for 60 min at 4 °C. The supernatant was collected and mixed with 4x NuPAGE™ LDS sample buffer (ThermoFischer, Waltham, USA), then heated at 70 °C for 10 min. After this, equal amounts of protein were given into the wells of sodium dodecyl sulfate–polyacrylamide gel electrophoresis (SDS-PAGE) gel (Mini-Protean TGX gel, Bio-Rad, Munich, Germany). Electrophoresis was performed for 1 h at 100–140 V. The gel was then blotted for 1 h at 100 V on a polyvinylidene difluoride (PVDF) membrane (Roche Applied Science, Mannheim, Germany). Upon completion of protein transfer, unspecific binding to the membranes was blocked by 1 h of incubation with 5% BSA dissolved in tris-buffered saline with Tween 20 (TBS-T). Then, the membranes were cut (to control the loading by GAPDH or α-actinin staining) and incubated with primary antibodies at 4 °C overnight. The antibodies were diluted in TBS-T with 5% BSA as follows: α-actinin (ALX-210-356-C050, Enzo Life Sciences, Farmingdale, NY, USA) 1:1000; GFP (MBL, Woburn, MA, USA) 1:200; hOCT2 (HPA008567, Sigma/Merck, Darmstadt, Germany) 1:250; GAPDH (1D4MMS-580S, BioLegend, San Diego, CA, USA) 1:5000. After this, the PDVF membranes were washed and incubated for 1 h with goat-anti-rabbit antibody (Dako, Hamburg, Germany) coupled with horseradish peroxidase (HRP) at a 1:10,000 dilution and washed again. Immunoreactive bands were detected with an imager system (ChemiDoc™ MP, Bio-Rad Laboratories, Hercules, CA, USA) by enhanced chemiluminescence using Lumi-Light Plus (Sigma/Merck, Darmstadt, Germany).

### 4.5. Measurement of OCT Function

The organic cation 4-(4-dimethylaminostyryl)-N-methylpyridinium (ASP^+^) was used as a fluorescent substrate of hOCT2 [48]. ASP^+^ is a well-known fluorescent substrate of OCT, as already shown in numerous publications [39,40,63]. Fluorescence was measured with a microfluorescence plate reader (Infinite F200, Tecan, Tecan Group Ltd., Crailsheim, Germany) using excitation at 450 nm and emission at 590 nm [64]. To evaluate hOCT2 function, ASP^+^ fluorescence was measured in cell lysates prepared after incubation with ASP^+^ from the apical or basolateral compartment. Transport measurements were performed at T = 37 °C. Before measurements, cells monolayers were washed with Ringer-like solution containing (in mM): NaCl 145, K_2_HPO_4_ 1.6, KH_2_PO_4_ 0.4, D-glucose 5, MgCl_2_ 1, calcium gluconate 1.3, and pH adjusted to 7.4 at 37 °C. After incubation, cells were washed with an ice-cold Ringer-like solution and lysed with 4% SDS in 10 mM Tris-HCl (pH 7.4). ASP^+^ concentration in cell lysates was quantified by a standard curve, where known ASP^+^ concentrations were added to cell lysates. The protein content of lysed cells was determined by absorption measurements using PicoDrop Pico 1000 (PicoDrop, Saffron Walden, UK). ASP^+^ cellular content was expressed as µmol ASP^+^/mg protein. In preliminary experiments (see Figure 3 and Figure 4), optimal ASP^+^ concentration (20 µM) and incubation time (60 min) were established (using these experimental conditions the transport of ASP^+^ was in the linear concentration range). Specific hOCT2-mediated ASP^+^ uptake was calculated by the subtraction of uptake measured in the presence of a high (1 mM) cimetidine concentration, a high-affinity hOCT2 inhibitor, from total uptake.

The affinity of hOCT2 for ASP^+^ was determined by saturation experiments of specific ASP^+^ uptake determined in the presence of increasing ASP^+^ concentrations (0–50 µM). In further experiments, regulation of hOCT2-mediated transport was evaluated by the co-incubation of ASP^+^ with 1 nM AII, or 1 µM DOG (a PKC activator, this concentration is known to be able to regulate OCT-activity [65]), or 40 µM rottlerin (a PKC inhibitor), or 5 µM BAPTA-AM (a Ca^2+^ chelator, this concentration efficiently chelates intracellular Ca^2+^ and does not compromise cell viability in MDCK cells [66]), alone or in combination. Solvents (DMSO or ethanol) at the concentration used in the regulation experiments did not change the ASP^+^ uptake (not shown). Cell cultures were grown on filters inserted in 24 or 12 well plates until 80–90% confluence was reached. Experiments were performed with cells from passages 20–45.

### 4.6. Cell Viability Test

The CDDP cytotoxicity was measured using a modified MTT test [67]. The MDCK cells grown to confluency on filters were incubated for 4 h at 37 °C with 100 µM CDDP (Teva Pharm, Ulm, Germany) dissolved in a Ringer-like solution and added to the apical or to the basolateral compartment. A CDDP concentration of 100 µM was used since it has already been shown that this CDDP concentration clearly interacts with hOCT2 function [8]. In some experiments, CDDP was incubated together with 1 nM AII. The CDDP solution was then removed, and the cells were further grown in fresh medium for 48 or 72 h. Afterward, the cells were incubated with 10 µL MTT (Sigma/Merck, Darmstadt, Germany) solution containing 5 mg/mL of the dye for three hours at 37 °C. Then, MTT was removed, and the cells were lysed with a solution containing 10% (*w*/*v*) SDS and 40% (*v*/*v*) dimethylformamide. Lysates were transferred to a new microtiter plate and after 90 min, absorption was measured at 570 nm in a multiplate reader (Tecan infinite m200, Tecan, Tecan Group Ltd., Crailsheim, Germany).

### 4.7. Permeability Assay

MDCK cells were seeded on filter as described above and cultivated with medium lacking phenol red. The same volumes were used for the apical and basolateral chambers. The cells were cultured until a confluent monolayer developed and a stable value of transepithelial electrical resistance (around 100–150 Ω/cm^2^, not shown) was reached. The medium in the apical chamber was replaced with the same medium containing 1 mg/mL FITC-dextran 70 kDa (Sigma/Merck, Darmstadt, Germany) and further incubated in the dark. Aliquots of medium (50 μL) were taken from the basolateral chamber after 10, 30, 180, 240 min and 72 h and replaced with fresh medium. The aliquots were transferred to 96-well plates and fluorescence was measured in the microplate reader described above using excitation at 492 nm and emission at 518 nm. The dextran concentration in the basolateral compartment was calculated using a standard curve from 0 to 50 µg dextran/mL.

### 4.8. Statistical Analysis

Data were analyzed using GraphPad Prism, Version 9.0 (GraphPad Software, Inc., San Diego, CA, USA). Data presented in this work are expressed as mean values ± SEM, with (*n*) referring to the number of replicates and N referring to the number of independent experiments. K_m_- and V_max_-values were obtained by a non-linear sigmoidal concentration-response curve fitting. When it is indicated, unpaired *t*-test and ANOVA test with posthoc Tukey were applied to prove statistical significance (*p* < 0.05).

## Figures and Tables

**Table 1:** Primers used for cloning (5′-3′).

Cloning of hOCT2 into pEGFP-N3:	
Forward:	CTC AGA TCT CGA GCT ATG CCC ACC ACC GTG GAC GAT
Reverse:	CGG GAT GGA TCC GTT CAA TGG AAT GTC TAG TTT
Cloning of hOCT2-GFP into pQCXIH:	
Forward:	GAT GCG GCC GCA TGC CCA CCA C
Reverse:	AAG CGG CTT CGG CCA GTA ACG TTA
Cloning of GFP into pQCXIH:	
Forward:	GAT GCG GCC GCA TGG TGA GCA AG
Reverse:	GCT TAA TTA ACT TGT ACA GCT CGT CCA TGC

## Data Availability

Not applicable.

## Supplementary Materials

The following supporting information can be downloaded at: https://www.mdpi.com/article/10.3390/ijms232415866/s1.
