# Supplementary material for: Exacerbation of Cisplatin Cellular Toxicity by Regulation of the Human Organic Cation Transporter 2 through Angiotensin II
Source: Int J Mol Sci. 2022 Dec 14;23(24):15866. doi: 10.3390/ijms232415866 (PMC9779897; doi:10.3390/ijms232415866)
Supplement: Supplementary file 1 [file ijms-23-15866-s001.zip › ijms-1992943-supplementary.pdf]

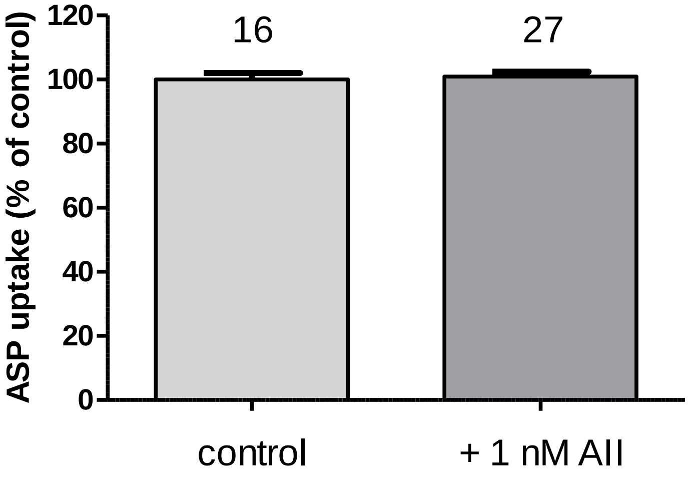

Supplementary material Figure S1: This figure shows the ASP^+ uptake measured in human embryonic kidney cells (HEK293 cells) cells stably transfected with hOCT2. Incubation for 10 minutes with 1 nM angiotensin II (AII) did not change the uptake rate of 1 μM ASP^+ compared with control measurements, which were set to 100%. The numbers on the columns show the number of replicates measured in at least 3 independent experiments. The experiments were performed as previously described in [1]

1. Frenzel; Köppen; Bauer; Karst; Schröter; Tzvetkov; Ciarimboli Effects of Single Nucleotide Polymorphism Ala270Ser (Rs316019) on the Function and Regulation of HOCT2. *Biomolecules* **2019**, *9*, 578, doi:10.3390/biom9100578.
